# Does fluralaner (Exzolt^®^ 5%) affect the enzootic stability of tick fever in beef calves subjected to strategic tick control in a tropical region?

**DOI:** 10.1186/s13071-025-07012-6

**Published:** 2025-10-17

**Authors:** Lidia Mendes de Aquino, Igor Maciel Lopes de Morais, Vanessa Ferreira Salvador, Luccas Lourenzzo Lima Lins Leal, Nathalia Braz Ribeiro, Luciana Maffini Heller, Gabriel Lopes Tamiozo, Raphaela Bueno Mendes Bittencourt, Nicolas Jalowitzki de Lima, Lorena Lopes Ferreira, Daniel de Castro Rodrigues, Tom Strydom, Siddartha Torres, Vando Edésio Soares, Caio Marcio de Oliveira Monteiro, Felipe da Silva Krawczak, Welber Daniel Zanetti Lopes

**Affiliations:** 1 Center for Veterinary Parasitology, School of Veterinary and Animal Science, Federal University of Goiás, Goiânia, Goiás Brazil; 2 Department of Preventive Veterinary Medicine, School of Veterinary and Animal Science, Federal University of Goiás, Goiânia, Brazil; 3 Department of Preventive Veterinary Medicine, School of Veterinary Medicine, Federal University of Minas Gerais, Belo Horizonte, Minas Gerais Brazil; 4 MSD Animal Health, São Paulo, Brazil; 5 MSD Animal Health, 20 Spartan RoadKempton Park 1619, Isando, South Africa; 6 Merck Animal Health, 2 Giralda Farms, Madison, NJ 07940 USA; 7 University of Brazil, São Paulo, Descalvado Brazil; 8 Department of Biosciences and Technology, Institute of Tropical Pathology and Public Health, Federal University of Goiás, Goiânia, Goiás Brazil

**Keywords:** Anaplasmosis, Babesiosis, Isoxazoline, *Rhipicephalus microplus*

## Abstract

**Background:**

Little is known about the impact of fluralaner on herd enzootic stability regarding tick fever pathogens (TFPs). This study aimed to assess whether implementing strategic control of *Rhipicephalus microplus* using fluralaner could influence the enzootic stability of beef herds regarding TFPs on a farm where this compound had never been previously used.

**Methods:**

One hundred *Bos taurus indicus* calves were divided into two groups of 50 animals each. One group underwent strategic tick control with pour-on fluralaner at 2.5 mg/kg (FLU), while the control group received a pour-on combination of fipronil 1.25 mg/kg + fluazuron 2.5 mg/kg (FIFLUA), a protocol used on the farm for 12 years. Calves were monitored from 25 to 241 days of age (weaning). During this period, in addition to acaricide treatments, tick counts and TFP diagnosis via indirect enzyme-linked immunosorbent assay (iELISA), quantitative polymerase chain reaction (qPCR), and blood smears were conducted in both groups.

**Results:**

FIFLUA animals received four acaricide treatments at 25, 60, 135, and 188 days of age, with intervals of 35, 75, and 53 days. FLU animals received three acaricide treatments, at 25, 135, and 188 days of age, with intervals of 110 and 53 days. The average tick count in the FLU group was significantly lower than in the FIFLUA group at 60 days (Kruskal–Wallis, *H* = 97.85, *df* = 1, *P* < 0.0001) and 241 days (*H* = 18.12, *df* = 1, *P* < 0.0001). *Anaplasma marginale* bacteremia was lower in the FLU group at 60 days (*H* = 3.98, *df* = 1, *P* = 0.0459). Serologically, enzootic stability for *Babesia bovis* was achieved at 135 and 188 days in the FLU and FIFLUA groups, respectively. For *B. bigemina*, over 75% of calves in both groups showed antibodies at 60 and 188 days, respectively. For *A. marginale*, enzootic stability was reached at 188 days (FLU) and 241 days (FIFLUA).

**Conclusions:**

Strategic tick control with fluralaner did not compromise the development of enzootic stability to *B. bovis*, *B. bigemina*, and *A. marginale*. Over 75% of FLU-treated calves showed seroconversion at 133, 60, and 188 days, respectively.

**Graphical Abstract:**

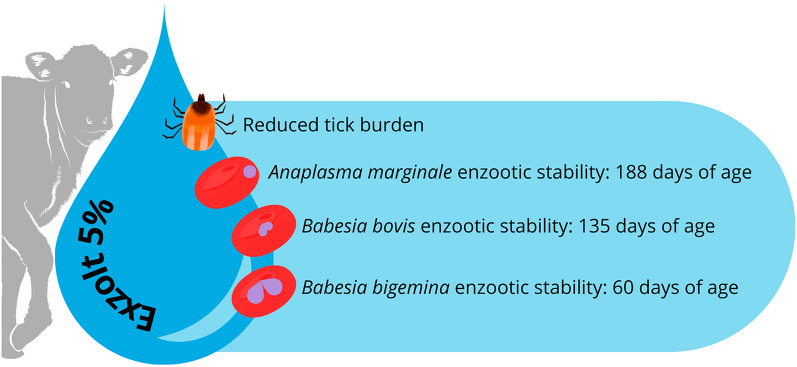

## Background

*Babesia bovis*, *Babesia bigemina*, and *Anaplasma marginale* are three of the most important pathogens affecting cattle, causing the syndrome popularly known as tick fever (TF). *Babesia* spp. are protozoa, while *A. marginale* is a rickettsia [1, 2], and both are listed as priority diseases in the Terrestrial Animal Health Code of the World Organization for Animal Health [3] because they cause significant losses to farmers. Studies of Holstein heifers in tropical regions show that each recurrence of TF pathogens presented by an animal between 3 and 7 months of age resulted in a reduction of 213.5 L of milk during the first lactation [4]. In addition, the percentage of herd mortality due to TF pathogens can range from 5% to 13% [5, 6].

In regions where *B. bovis*, *B. bigemina*, and *A. marginale* are endemic, cattle should be exposed to these three TF pathogens at a young age to achieve enzootic stability of the herd. This avoids clinical cases of babesiosis and anaplasmosis in adult cattle [7], from which clinical recovery is more difficult and mortality is higher [6]. The definition of enzootic stability was first established for *Babesia* spp. by Mahoney and Ross [8]. According to these authors, a herd reaches stable status for *Babesia* spp. if the animals achieve a serological prevalence ≥ 75% up to 9 months of age. After that, the animals tend to have no problems with babesiosis. The same concept has been extrapolated to *A. marginale* and was recently validated [9]. In tropical and subtropical regions, herd enzootic stability is directly related to cattle exposure to *Rhipicephalus microplus* [4]. In addition, the tick control product together with the treatment regimen used may be a factor determining whether cattle achieve enzootic stability against TF pathogens [10].

In this sense, the development of new acaricide molecules that are 100% effective, and how they are used, is generating concern in the field. In this regard, fluralaner stands out. It was first launched in Brazil in 2022 and achieved 100% efficacy against *R. microplus* [11]. The relationship between the use of fluralaner against this tick species and the enzootic stability of the herd for TF pathogens has already been studied in Holstein heifers raised on pasture in a tropical climate region [12]. In that study, the authors reported that the scheme adopted with fluralaner against the cattle tick did not negatively affect the transmission of TF pathogens to the calves. However, [12] highlighted the importance of carrying out further studies on other farms with cattle that are less challenged by *R. microplus*, such as *Bos taurus indicus*, to find out how the transmission of TF pathogens would behave in these animals when a strategic control program against the cattle tick with fluralaner is used. Given this gap, we aimed to determine whether strategic control of *R. microplus* with pour-on fluralaner could influence the enzootic stability of the herd for TF pathogens.

## Methods

### Experimental location

This experiment was carried out from October 2023 to May 2024 in a commercial cattle farm located in the municipality of Rio Verde, Goiás, Brazil. This is a tropical region where there are two well-defined seasons per year: the rainy summer (October to April), with average annual rainfall of 1541 mm, and the dry winter (May to September), with 150–200 mm of rainfall.

The farm has about 4500 head of cattle. The cows are 1/2 Angus × 1/2 Nelore and the calves, obtained by artificial insemination, are 1/2 Angus × 1/2 Nelore and 3/4 European × 1/4 Nelore. Depending on their reproductive status, cows stay on the farm until their fifth pregnancy on average. All male calves and a portion of the female calves are confined after weaning and slaughtered at approximately 14 months of age. A portion of the 1/2 Angus × 1/2 Nelore female calves remain on the farm after weaning, on average, until the fifth gestation. The cattle on this farm are infested with *R. microplus* and maintain contact with TF pathogens. Sporadic outbreaks of *A. marginale* occur in calves between 17 and 25 days of age [6]. After 4 months of age, pasture-raised animals no longer present clinical problems due to TF pathogens, suggesting an enzootic stability to TF pathogens in animals from this age onwards.

For the past 12 years, cattle tick control on this farm has been based on empirical criteria established by farm staff, with treatment based on inspection of animals to visually assess the presence of ticks. The tick treatment used is a pour-on formulation of fipronil 1.25 mg/kg + fluazuron 2.5 mg/kg (TickGard^®^, MSD Animal Health). Pour-on fluralaner at 2.5 mg/kg (Exzolt^®^ 5%, MSD Animal Health) had never been used on this farm before the start of this study.

### Animals, groups, treatments, and experimental period

The study used 100 male and female 3/4 European × 1/4 Nelore calves that had not been treated for endo- or ectoparasites since birth and were naturally infested with *R. microplus* at the beginning of the study. When these animals were an average of 25 days old, they were divided into two groups of 50 animals each: FIFLUA and FLU. The FIFLUA group was treated with a commercial pour-on formulation of fipronil 1.25 mg/kg + fluazuron 2.5 mg/kg (TickGard^®^, MSD Animal Health) and the FLU group with a pour-on formulation of fluralaner 2.5 mg/kg (Exzolt^®^ 5%, MSD Animal Health). The experimental period was based on the age of the calves. They were observed from 25 days of age until weaning, which occurred at an average age of 241 days. The criterion for allocating the animals to the groups was based on tick count/burden, age, and sex of the animals. After randomization, the groups were homogeneous in mean tick counts (FLU = 1.9 ± 2.5; FIFLUA = 1.8 ± 2.4), age in days (FLU = 25.3 ± 4.14; FIFLUA = 25.6 ± 5.24), and sex (FLU and FIFLUA = 23 females and 27 males each).

Throughout the experimental period (25 to 241 days of age), each group remained in a different paddock, with their respective mothers, practically identical in size and the availability of grass cover, and each group was kept separate from the other throughout the experimental period. The calves were fed maternal whole milk from cows during the entire period, plus *Brachiaria brizantha* and water ad libitum.

### Strategic treatments against *R. microplus* on calves

The FIFLUA group served as a control. The commercial product used to treat the animals in this group was already in use on the farm. In addition, the animals receiving the treatment adopted by the farm no longer presented clinical cases of TF pathogens after approximately 2 to 4 months of age, suggesting enzootic stability for TF pathogens. The first treatment was performed on all animals of this group at 25 days of age. Therefore, the treatment criteria for the FIFLUA group corresponded to the practice of the farm staff. When this happened, all 50 animals in the group were treated.

The first treatment of the FLU group was performed at the beginning of the study (animals at 25 days of age). Calves in the FLU group were re-treated with fluralaner when more than 30% of the batch (15/50) had *R. microplus* infestations < 4 mm in length, according to the method described by [13], independent of the number of ticks per animal. In each case, all 50 animals were treated. Visual inspection of animals in the FLU group was performed at weekly intervals throughout the experiment, always in the pasture.

The cows in each group received the same acaricide as the calves, but only once at the beginning of the study. In addition, the other activities described below did not apply to the cows.

Before each treatment in both groups, the animals were weighed to calculate the acaricide dose. The scales used to weigh the animals were previously tested with a known weight. The commercial products were administered topically using a 10-mL syringe graduated in 0.2-mL increments. Therefore, the calculated dose was rounded down; for example, an animal weighing 107 kg that would have received 10.7 mL of a pour-on product received 10.6 mL. Cattle treated with the pour-on formulations were not exposed to rain for 48 h after each treatment.

### Tick counts and evaluation of the enzootic stability of TF

Female *R. microplus* ticks (between 4.5 and 8 mm in length) present on the left side of each animal (without multiplying by two) were counted on all calves [14] at the beginning of the study (25 days of age) to divide the animals into groups. Tick counts were then performed on both groups (FIFLUA and FLU) at average ages of 60, 135, 188, and 241 days (weaning).

The enzootic stability of the herd was assessed by indirect enzyme-linked immunosorbent assay (iELISA) on the same days as the tick counts, when the animals were 25, 60, 135, 188, and 241 days old. For this purpose, the serum or blood of 15 animals per group was randomly selected and evaluated throughout the experimental period. Immunoglobulin G (IgG) antibodies against *A. marginale*, *B. bovis*, and *B. bigemina* were detected by iELISA according to the protocol described by [15, 16].

Quantitative polymerase chain reaction (qPCR) was used to determine the frequency with which the animals of each group was exposed to the TF pathogens. To detect the DNA of the three agents, blood samples (300 µl) were subjected to DNA extraction using the DNAeasy Blood and Tissue Kit (Qiagen, Valencia, CA, USA), following the manufacturer’s instructions. The extracted DNA was tested using two different qPCR approaches: a monoplex qPCR assay targeting *A. marginale* and a duplex qPCR assay targeting *B. bovis* and *B. bigemina*. The monoplex qPCR assay targeted a fragment of the major surface protein 1b (msp1b) gene of *A. marginale* [17]. The duplex qPCR assay targeted the mitochondrial cytochrome B (cytB) gene of *B. bovis* and *B. bigemina*, as described by [18], with modifications to the quenchers (Table 1). Each qPCR run included a negative control (PCR-grade water, Sigma-Aldrich, St. Louis, MO, USA) and an appropriate positive control sample (DNA from *A. marginale*, *B. bovis*, or *B. bigemina*).

**Table 1:** Oligonucleotides (primers and probes) used in the present study

Target gene/specificity	Primer/probe: sequence 5′–3′	Amplicon size (bp)	References
qPCR			
*msp 1b*/*A. marginale*	AM-For: TTGGCAAGGCAGCAGCTT AM-Rer: TTCCGCGAGCATGTGCAT AM-Pb: 5′ 6FAM-TCGGTCTAACATCTCCAGGCTTTCAT-3′ QSY	95 bp	Carelli et al. 2007
*cytB/B. bovis*	BOT-F: TGTTCCTGGAAGCGTTGATTC BOT-R: CCAACCCATATTGACTTCAGC BOT-P: 5′ VIC-TGAATGTGTAATTAGAGTGC-3′ MGB-NFQ	n.i.	Criado-Fornelio et al. 2009
*cytB*/*B. bigemina*	BIT-F: TTATGTTCCAGGAGATGTTGA BIT-R: CCCAACCCATATTAACCTCAGT BIT-P: 5′ FAM-CGAATGTGTTATCAGAGTAT- 3′ MGB-NFQ	n.i.	Criado-Fornelio et al. 2009
PCR			
*cytB*/Mammals	L14841-F: CCATCCAACATCTCAGCATGATGAAA H15149-R: GCCCCTCAGAATGATATTTGTCCTCA	359 bp	Kocher et al. 1989

*n.i.* not informed, *bp* base pairs

All qPCR assays were performed using TaqMan^®^ Environmental Master Mix 2.0 (Applied Biosystems^®^, Foster City, CA, USA) according to the manufacturer’s recommendations, in a StepOnePlus™ Real-Time PCR System thermal cycler (Applied Biosystems^®^, Foster City, CA, USA). The thermal cycling conditions consisted of an initial DNA polymerase activation at 95 °C for 10 min, followed by 40 cycles of denaturation at 95 °C for 15 s and annealing-extension at 60 °C for 1 min. For all assays, lower CT values corresponded to a higher amount of starting template, and a negative result was defined as having a CT value > 35 cycles. Negative samples were further tested using a conventional PCR protocol targeting a 359 bp fragment of the mammalian mitochondrial cytochrome B (cytB) protein gene [19] to validate the DNA extraction procedure. When a sample failed to produce any product in this PCR assay, it was excluded from further analysis. PCR products were stained with SYBR Safe (Invitrogen, Thermo Fisher Scientific, Waltham, MA, USA) according to the manufacturer’s recommendations and visualized by 1.5% agarose gel electrophoresis under an ultraviolet transilluminator.

According to [8] and [9], a herd is considered enzootically stable for *Babesia* spp. and *A. marginale* if more than 75% of the animals are positive by serology for these pathogens. Therefore, in this study, enzootic stability was considered for each of the three TF agents when more than 75% of the animals in a group were positive by serology, while qPCR was used to confirm the frequency with which the animals of each group were exposed to the TF pathogens.

### Blood smears: *Babesia* spp. parasitemia and *A. marginale* bacteremia

Blood smears were performed throughout the experiment to calculate *Babesia* spp. parasitemia and *A. marginale* bacteremia in the calves. However, this technique was not considered for the evaluation of enzootic stability due to its low sensitivity. Parasitological diagnosis was determined by blood smears for TF pathogens obtained from all animals in both groups at 25, 60, 135, 188, and 241 days of age. Blood was collected from the tip of the calves’ tails, stained with Giemsa, and examined under a microscope at 1000× magnification. The percentage of parasitemia or bacteremia for TF pathogens was determined using the method described by [20, 21].

### Salvation treatment of the animals during the study against TF pathogens

To ensure animal welfare during the entire experimental period, if any calf showed clinical signs of TF pathogens, this animal received salvation treatment with diminazene 3.5 mg/kg (Ganazeg^®^, Elanco Animal Health [22]) plus 7.5 mg/kg of enrofloxacin intramuscularly (Knetomax^®^, Elanco Animal Health [23]). These signs involved the head, ears, and eyelids drooping, combined with the fact that the animal in question was more isolated from the herd, or that the animal was unable to keep up with the rest of the herd while the group was moving. Each time this happened, the animal’s identification and date were recorded, and a blood smear was performed to confirm the diagnosis of which TF agent was involved.

### Statistical analyses

The data for tick counts and serological tests did not meet the assumptions of normality, homogeneity of variance, residuals, and randomness, even after transforming the data into log(count + 1). Therefore, the experimental groups were compared using the Kruskal–Wallis test.

All statistical procedures were performed using the Statistical Analysis System [24], version 9.4 (2016). Differences were considered statistically significant when *P* < 0.05.

## Results

During the experimental period, when the calves were between 25 and 241 days old, and according to the criteria established for each group, the calves in the FIFLUA group received four acaricide treatments. The treatments took place at 25, 60, 135, and 188 days of age, with an interval of 35, 75, and 53 days between applications, respectively (Table 2). Animals in the FLU group received three acaricide treatments at 25, 135, and 188 days of age, with respective intervals of 110 and 53 days between treatments (Table 2).

**Table 2:** Number of animals infested with ticks < 4 mm in length between the legs and dewlap region, and mean count of *Rhipicephalus microplus* (female between 4.5 and 8 mm in length) on the left side of the body of animals subjected to different tick control schemes

Age of the animals (days)	Number of animals with ticks < 4 mm in length (%)		Tick counts (females 4.5–8 mm in length)	
			Treatments/mean and standard deviation*	
	FIFLUA	FLU	FIFLUA	FLU
25 ^αβ^	17/50 (34.0%)	16/50 (32.0%)	1.8 ± 2.4 ^Ac^	1.9 ± 2.5 ^Ac^
60 ^α^	50/50 (100.0%)	0/50 (0.0%)	28.3 ± 23.2 ^Aa^	0.0 ± 0.0 ^Bd^
135 ^αβ^	22/50 (44.0%)	24/50 (48.0%)	1.3 ± 2.4 ^Bc^	6.8 ± 7.9 ^Aa^
188 ^αβ^	49/50 (98.0%)	39/50 (78.0%)	2.3 ± 3.1 ^Abc^	4.2 ± 5.8 ^Ab^
241	40/50 (80.0%)	12/50 (24.0%)	6.3 ± 5.3 ^Ab^	2.2 ± 3.4 ^Bc^

* Values ​​followed by the same uppercase letter in the row and lowercase letter in the column do not differ from each other

*FIFLUA* fipronil 1.25 mg/kg + fluazuron 2.5 mg/kg, pour-on,  *FLU* fluralaner 2.5 mg/kg, pour-on

*α* = when all 50 animals in FIFLUA group received fipronil + fluazuron

*β* = when all 50 animals in FLU group received fluralaner

The tick burden in the FLU-treated group was lower than in the FIFLUA group at 60 (Kruskal–Wallis *H*-test, *H* = 97.85, *df* = 1, *P* < 0.0001) and 241 (Kruskal–Wallis *H*-test, *H* = 18.12, *df* = 1, *P* < 0.0001) days of calf age. On the other hand, animals in the FIFLUA group had lower tick burdens than those in the FLU group at 135 days of age (Kruskal–Wallis *H*-test, *H* = 20.72, *df* = 1, *P* < 0.0001) (Table 2).

Bacteremia by *A. marginale* in the FLU group was lower than in the FIFLUA group at 60 days of age (Kruskal–Wallis *H*-test, *H* = 3.98, *df* = 1, *P* = 0.0459) (Table 3). Also, by blood smears, one animal from the FLU group at 135 days of age and one from the FIFLUA group at 60 days of age showed *B. bigemina* infection. No other calves were diagnosed with *Babesia* spp. by blood smears on the other dates, so it was not possible to calculate the parasitemia of the protozoan in question for the different groups.

**Table 3:** Bacteremia of *Anaplasma marginale* in beef calves subjected to different control schemes against *Rhipicephalus microplus*

Age of animals (days)	Treatments/mean and standard deviation bacteremia*	
	FIFLUA	FLU
25	0.020 ± 0.061 ^Ac^	0.010 ± 0.042 ^Ab^
60	0.319 ± 0.633 ^Aa^	0.106 ± 0.283 ^Ba^
135	0.161 ± 0.308 ^Ab^	0.085 ± 0.199 ^Aa^
188	0.049 ± 0.127 ^Abc^	0.115 ± 0.256 ^Aa^
241	0.056 ± 0.125 ^Abc^	0.068 ± 0.120 ^Aa^

* Values ​​followed by the same uppercase letter in the row and lowercase letter in the column do not differ from each other

*FIFLUA* fipronil 1.25 mg/kg + fluazuron 2.5 mg/kg, pour-on

*FLU* fluralaner 2.5 mg/kg, pour-on

**Fig. 1:**
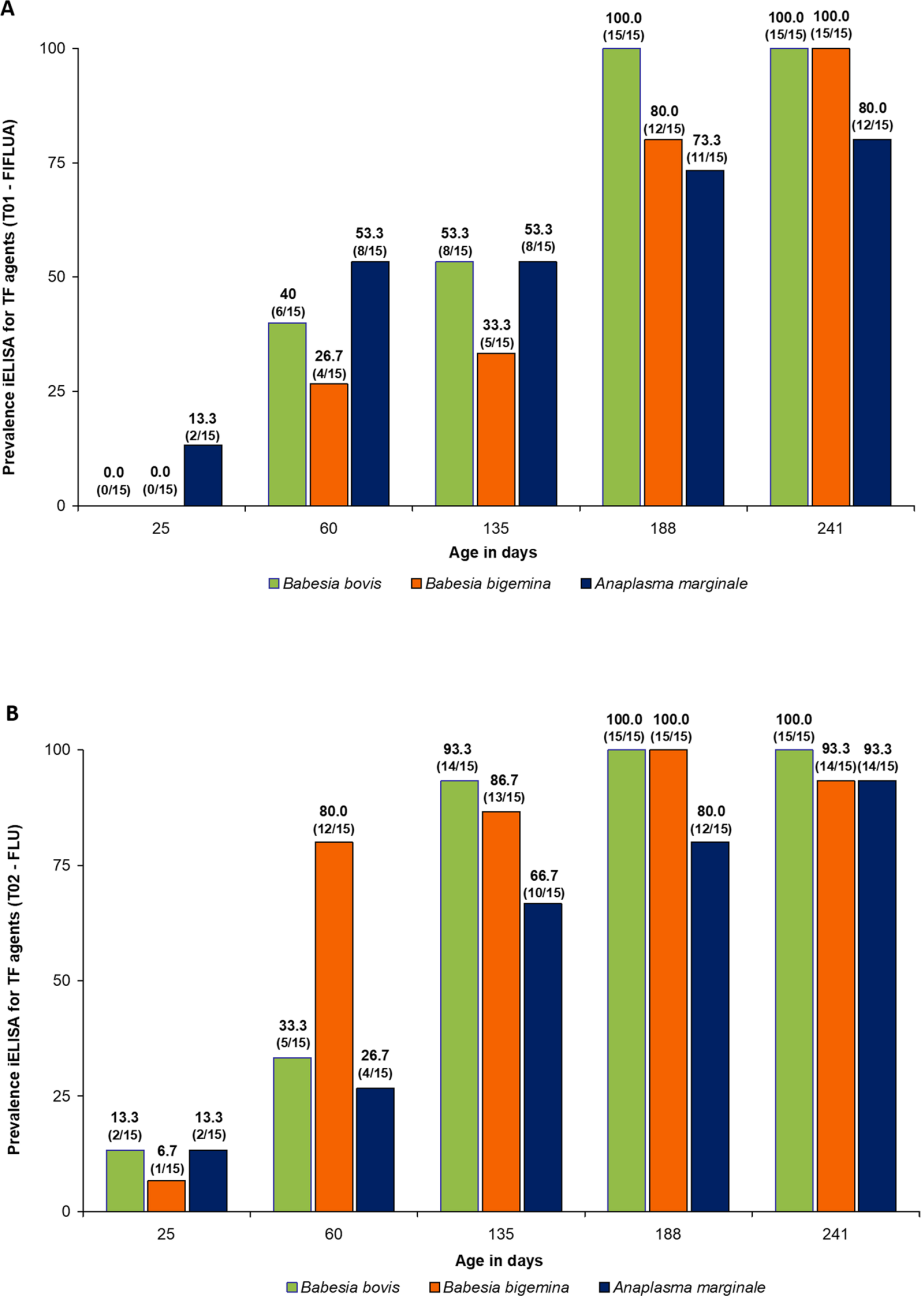
Number of animals positive for antibodies against *Babesia bovis*, *Babesia bigemina*, and *Anaplasma marginale* detected by serology (iELISA). The animals were subjected to different control schemes: **A** FIFLUA: fipronil 1.25 mg/kg + fluazuron 2.5 mg/kg, pour-on; **B** FLU: fluralaner 2.5 mg/kg, pour-on) against *Rhipicephalus microplus* from 25 to 241 days of life

Serologically, the enzootic stability status for *B. bovis* was reached in the FLU group at 135 days of age, while in the FIFLUA group it was reached at 188 days of age. As for *B. bigemina*, antibodies against this species of *Babesia* spp. were detected from 60 days of age in more than 75% of the calves in the FLU group, while in the FIFLUA group this occurred from 188 days of age. Regarding *A. marginale*, the calves treated with FLU reached the enzootic stability status for this rickettsia from 188 days of age, while the group of animals treated with FIFLUA showed the same status from 241 days of age (weaning) (Fig. 1A, B). Considering the experimental period, the average antibody titres against the three TF pathogens (*B. bovis*, *B. bigemina*, and *A. marginale*) of the animals treated with FLU were higher than the average antibody titres found in the calves treated with FIFLUA (Kruskal–Wallis *H*-test, *H* = 15.63, *df* = 1, *P* < 0.0001).

**Fig. 2:**
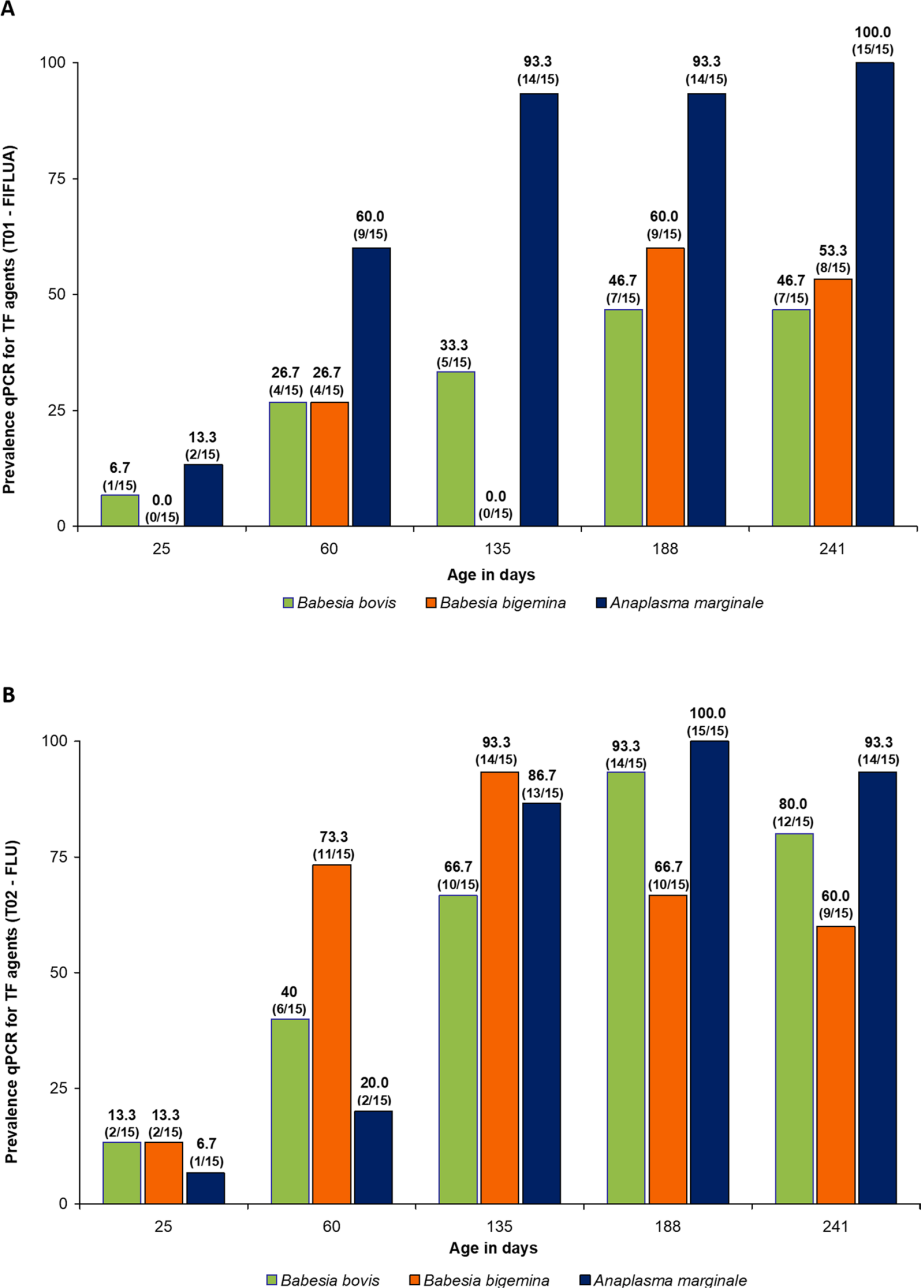
Number of bovines positive for *Babesia bovis*, *Babesia bigemina*, and *Anaplasma marginale* DNA by molecular assay (qPCR). The animals were subjected to different control schemes: **A** FIFLUA: fipronil 1.25 mg/kg + fluazuron 2.5 mg/kg, pour-on; **B** FLU: fluralaner 2.5 mg/kg, pour-on) against *Rhipicephalus microplus* from 25 to 241 days of life

According to qPCR, the frequency of *B. bovis* infection in the FIFLUA group ranged from 6.7% (1/15) to 46.7% (7/15) at 25 and 241 days of age, respectively. For *B. bigemina*, the frequency of infection in animals in this group ranged from 0.0 (0/15) to 26.7% (4/15) between 25 and 133 days of age, reaching 60% (9/15) and 53.3% (8/15) at 188 and 241 days of age, respectively. Against *A. marginale*, the presence of DNA from this rickettsia was found in 13.3% (2/15) at 25 days of age to 100% (15/15) of the animals at 241 days of age (Fig. 2A).

In the FLU-treated group, the presence of *B. bovis* DNA was detected in 13.3% (2/15), 40% (6/15), 93.3% (14/15), and 80.0% (12/15) of the calves at 25, 60, 188, and 241 days of age, respectively. For *B. bigemina*, 13.3% (2/15) of the calves were positive in the qPCR at the beginning of the study, increasing to 93.3% (14/15) at 135 days of age and then to 60% (9/15) at weaning. As for *A. marginale*, the presence of this rickettsial DNA occurred in 6.7% (1/15) of the calves at an average age of 25 days, increased to more than 75% at 135 days of age, and remained so until the end of the study (241 days—weaning) (Fig. 2B).

Five calves from the FIFLUA group required salvation treatment with enrofloxacin + diminazene at 60 days of age. Parasitological diagnosis by blood smears revealed the presence of red blood cells infected with *A. marginale*, with bacteremia ranging from 1.7% to 3.2%. Due to this incident, the farm manager decided to treat all the other calves in this group (FIFLUA) with imidocarb dipropionate (1.2 mg/kg Imizol^®^, MSD Animal Health).

## Discussion

This study provides important practical results regarding the strategic control of cattle ticks with fluralaner and its effect on the enzootic stability of TF pathogens in crossbred beef calves. The 100% efficacy of pour-on fluralaner at 2.5 mg/kg (Exzolt^®^ 5% MSD Animal Health) against *R. microplus* in dairy and beef cattle has already been demonstrated in previous studies [11, 25–27]. Despite the efficient control provided by fluralaner, there is concern about how to use it for strategic control against the cattle tick. Depending on the situation, the question is whether the use of this highly effective product could affect the enzootic stability of the herd for TF pathogens. This issue has already been studied in dairy cattle (*Bos taurus taurus*) raised in a tropical climate region, where it was observed that strategic control of bovine ticks with fluralaner did not interfere with the enzootic stability of the herd for TF pathogens [12].

It is well known that the level of challenge of crossbred cattle by *R. microplus* is lower compared to taurine animals, thus requiring fewer chemical treatments to control this tick species in crossbred cattle [28–30]. However, based on the results obtained in the present study, it was possible to demonstrate that strategic control with fluralaner did not affect the enzootic stability of TF pathogens in crossbred animals raised on pasture in a tropical climate region. For the FLU group, enzootic stability by iELISA was achieved from 133, 60, and 188 days of age for *B. bovis*, *B. bigemina*, and *A. marginale*, respectively, according to the recommendations described by [8] and [9]. In the control group (FIFLUA), treated at the farm manager’s discretion, the same stability statuses were achieved at 188, 188, and 241 days of age for *B. bovis*, *B. bigemina*, and *A. marginale*, respectively.

It is important to note that the tick re-treatment criterion used for FLU in this study was the same as that described by [12] and [13]. This method consists of treating animals when more than 30% of the herd has ticks < 4 mm in length on the crotch and dewlap. In other words, instead of determining the day of re-treatment, it is based on the re-infestation of the animals. This allows the calves to come into contact with the bovine ticks and thus with the TF pathogens. Another positive aspect of this criterion is that in practice, the interval between treatments can be increased. Based on the efficacy results demonstrated by fluralaner in different tick populations, from a technical point of view, to interrupt the life cycle of the bovine tick, re-treatments should be carried out at intervals of 42 days [11, 25, 26]. In the present study, using the method described by [13], the interval between re-treatments was up to 105 days. In *B. t. taurus* cattle, using the same method, [12] and [25] obtained an average interval between treatments of 56 days between applications. In practice, this could mean fewer treatments throughout the year, resulting in less selection pressure on the tick population for resistance to fluralaner.

Another point to consider is that the increase in the interval between treatments generally occurs in formulations where the susceptibility of the *R. microplus* population to a given active ingredient is high (product efficacy close to 100%, as in this study). In situations where the resistance process of the *R. microplus* population is already more advanced for a given chemical class, such as in the case of fipronil and fluazuron in tropical climates, there is a tendency for the interval between acaricide applications to decrease from those originally specified [13, 29].

According to the results of the present study and those of [12] in a tropical climate, it can be concluded that high tick loads are not necessary for immunity to TF pathogens. In the present study, the average number of ticks (4.5–8 mm engorged females) found on calves treated with FLU was less than 6.8 during approximately 200 days. In the study conducted by [12], the infestation during 175 days was less than 2 ticks per animal. It should be noted that these results can particularly occur in tropical climates, where *R. microplus* completes up to five or six generations per year [31–34], and consequently, cattle contact with TF pathogens is more frequent. On the other hand, this scenario may be different in subtropical climates, where the same tick species completes only three annual generations [35, 36].

Keeping cattle with a relatively "low" tick infestation is desirable because *A. marginale* bacteremia can increase as *R. microplus* infestation rises in the animals. In the present study, when *A. marginale* bacteremia was observed in FIFLUA calves, it increased at 60 days of age, when the cattle tick infestation also increased in these animals. This fact triggered clinical cases of anaplasmosis in five calves of this group (FIFLUA) at this age. On the other hand, the application of FLU to calves at 25 days of age kept the number of ticks in these animals at zero at 60 days of age, and no calf in this group showed clinical cases of TF pathogens. This is another important result that should be considered in practice. On the same farm where this study was conducted, sporadic outbreaks of anaplasmosis occur, resulting in calf mortality between 18 and 25 days of age [6]. When these outbreaks were described by [6], calves did not receive tick control treatment until 40 days of age. Younger animals have higher tick infestation compared to adult cattle, possibly because they have less immunological competence [37], and in some way the degree of tick infestation may interfere with bacteremia by *A. marginale*, further exacerbating the clinical status of calves in this age group for TF pathogens.

It is important to emphasize that the results of the present study should not be extrapolated to all regions. Furthermore, since the presence of *R. microplus* is a determining factor for the occurrence of TF infections in cattle in tropical climates [4, 38], periods of unfavorable weather conditions that may affect the dynamics of *R. microplus* throughout the year, combined with the treatment of animals with fluralaner, may produce enzootic stability results different from those found in the present study, so these aspects must be taken into account. In any case, the present study shows important practical results that should be disseminated among veterinarians and field technicians. This can prevent losses to producers caused by TF pathogens.

## Conclusions

The strategic tick control with pour-on fluralaner, using the re-treatment criteria described in the current study, did not negatively affect the enzootic stability of crossbred beef calves for *B. bovis*, *B. bigemina*, and *A. marginale*. Serologically, more than 75% of the group treated with fluralaner produced antibodies against *B. bovis*, *B. bigemina* and *A. marginale* at 133, 60, and 188 days of age.

## Data Availability

Data supporting the main conclusions of this study are included in the manuscript.
